# COMMONALITIES IN REHABILITATION DATA ACROSS DIVERSE HEALTH CONDITIONS: A COMPARISON OF SEVEN LARGE EUROPEAN DATABASES

**DOI:** 10.2340/jrm.v58.45495

**Published:** 2026-03-23

**Authors:** Carlotte KIEKENS, Helena BURGER, Paolo CAPODAGLIO, Maria G. CERAVOLO, Esther JANSSEN, Greta JURENAITE, Calogero MALFITANO, Federico PENNESTRI, Ruud SELLES, Gianluca M. TARTAGLIA, Stefano NEGRINI, the PREPARE Group

**Affiliations:** 1 IRCCS Galeazzi – Sant’Ambrogio Hospital, Milan, Italy; 2 University Rehabilitation Institute, Ljubljana, Slovenia; 3 University of Ljubljana, Faculty of Medicine, Ljubljana, Slovenia; 4 Department of Biomedical, Surgical and Dental Sciences, University of Milan, Milan, Italy; 5 IRCCS Istituto Auxologico Italiano, Milan, Italy; 6 Department of Experimental and Clinical Medicine, Marche Polytechnic University, Ancona, Italy; 7 IQ Health, Radboud University Medical Center, Radboud Institute for Health Sciences, Nijmegen, The Netherlands; 8 School of Allied Health, HAN University of Applied Sciences, Nijmegen, The Netherlands; 9 Department of Orthopaedic Surgery, Viecuri Medisch Centrum, Venlo, the Netherlands; 10 ISICO (Italian Scientific Spine Institute), Milan, Italy; 11 Department of Biomedical Sciences for Health, University of Milan, Milan, Italy; 12 Azienda di Servizi alla Persona Istituti Milanesi Martinitt e Stelline e Pio Albergo Trivulzio, Milan, Italy; 13 Department of Rehabilitation Medicine, Erasmus MC, University Medical Center Rotterdam, Rotterdam, The Netherlands; 14 Department of Plastic and Reconstructive Surgery, Erasmus MC, University Medical Center Rotterdam, Rotterdam, the Netherlands; 15 Policlinico Hospital, Fondazione IRCCS Cà Granda Ospedale Maggiore Policlinico, Milan, Italy

**Keywords:** decision support systems, clinical, precision medicine, predictive learning models, rehabilitation

## Abstract

**Objective:**

To investigate whether rehabilitation data share common characteristics across different health conditions and care settings within the EU Horizon PREPARE project.

**Design:**

Qualitative content analysis, with a comparative study of existing clinical databases.

**Subjects/Patients:**

Individuals with hand and wrist disorders, idiopathic scoliosis, intermittent claudication, lower limb amputation, Parkinson’s disease or Parkinsonism, hip or knee replacement, and temporomandibular disorders.

**Methods:**

Seven rehabilitation-oriented clinical databases were analysed using the International Classification of Functioning, Disability and Health (ICF) framework. Variables were categorized as outcomes, modifiers, or baseline measurements. Commonalities and differences across data domains were identified through iterative consensus meetings among PREPARE partners.

**Results:**

Substantial heterogeneity was observed in data type and depth. Pain and quality of life were the most commonly reported outcomes, whereas discharge status and participation-related measures were rarely reported. The most prevalent modifiers were pharmacological treatments, orthoses or prostheses, and exercise-based interventions. All databases reported baseline information on diagnosis, anthropometry, and demographics; however, assessments of gait autonomy and daily activities were inconsistently documented.

**Conclusion:**

Despite some overlapping domains, rehabilitation data collection remains fragmented and predominantly focused on biomedical aspects. Greater standardization and systematic inclusion of psychosocial and contextual variables are needed for robust predictive modelling and personalized rehabilitation.

## LAY ABSTRACT

Rehabilitation is vital to healthcare, yet data collection remains inconsistent. Although numerous clinical databases exist, little is known about the similarities and differences in rehabilitation data across illnesses and settings. In this study, we analysed 7 European rehabilitation databases to identify what information is typically recorded and what is missing. We found that diagnosis, pain level, and overall well-being are often measured, whereas details on how well a patient can walk, perform self-care, or participate in social life after leaving hospital are often lacking. These gaps make it difficult for healthcare providers to compare results or create the best recovery plans for individuals. By standardizing the information we collect, we can improve decision-making and ensure every patient receives care tailored to their specific needs.

Rehabilitation plays a crucial role in optimizing health outcomes and promoting the well-being of populations. According to the World Health Organization (WHO), approximately 2.6 billion individuals could benefit from rehabilitation services. This need is expected to rise due to ageing populations and an increase in chronic diseases (1, 2). However, access to rehabilitation remains insufficient and inequitable, especially in low- and middle-income countries, where over 50% of those requiring rehabilitation services do not receive them (3, 4). In a healthcare context, rehabilitation is defined as a “multimodal, person-centred, collaborative process, including interventions targeting a person’s capacity (by addressing body structures, functions, and activities/participation) and/or contextual factors related to performance with the goal of optimizing the functioning of persons with health conditions currently experiencing disability or likely to experience disability, or persons with disability” (5). In numerous health conditions, rehabilitation should be considered either at or immediately after the acute onset, and it continues over time in the post-acute and eventually chronic phases (6). Rehabilitation is cross-sectional to many medical fields and shares commonalities between these fields (7). Nevertheless, scarce attention has been given to checking how specific commonalities are implemented in different fields. The EU Horizon PREPARE (Personalised REhabilitation via Novel AI PAtient StRatification StratEgies) project, a European initiative aimed at developing, validating, and implementing robust, clinically relevant, and data-driven computational prediction and stratification tools for the whole field of rehabilitation, offers an important opportunity to evaluate the field cross-sectionally (8). PREPARE focuses on the complexity of rehabilitation, stemming from factors such as multiple health conditions, varied patient experiences, and socioenvironmental contexts (5). The following issues, particular to rehabilitation, are taken into account: (a) functioning, the primary rehabilitation outcome and third health indicator (alongside morbidity and mortality), is multifaceted and mostly individually defined (9); (b) rehabilitation treatment is often complex as it requires more than 1 intervention at a time and a multiprofessional and interdisciplinary approach; (c) people in need of rehabilitation are often multimorbid and require shared decision-making to achieve personalized goals; (d) treatments cross over different healthcare phases; (e) outcomes are not only influenced by clinical indicators but also depend on socioeconomic, behavioural, and living condition indicators and availability of services. For many rehabilitation patients, the most effective treatment approach is multimodal, combining various therapeutic methods to address biological, psychological, and social factors in a coordinated and supportive manner.

In PREPARE, the development of prediction and stratification models is based on large datasets from 7 rehabilitation services in 3 European countries, spanning multiple health conditions, out of a total of 9 pilot cases. These databases were selected after a call shared with the national delegates of the European Society of Physical and Rehabilitation Medicine, for large rehabilitation databases in their countries. The first step of PREPARE was to analyse the commonalities and differences of the variables included in these databases, providing an overview of routinely collected clinical rehabilitation data. These variables demonstrate the attention paid to different topics in rehabilitation patients with varying health conditions. This paper aims to report the PREPARE findings, providing a first answer to the research question: Are there similarities in routine clinical rehabilitation variables collected from patients with different health conditions?

## METHODS

### Study design

We conducted a qualitative study to compare datasets and rehabilitation processes across 7 European clinical databases. We gradually defined and progressively modified the methodology as needed through a series of consensus meetings every 15 days, where results were presented incrementally. The meetings systematically involved all paper authors, who represented the 7 clinical partners involved, as well as other PREPARE partners, including social scientists, engineers, lawyers, communication specialists, and administrators. Tools were developed and iteratively refined through pilot implementations, feedback from partners, and discussions during general assemblies. Final data validation involved interviews with clinical partners.

### Pilot case descriptions

Of a total of 9 pilot cases considered in PREPARE, 2 (people with hypertension and people operated on for spine disorders) were not focused directly on rehabilitation and were excluded from this analysis. The 7 included pilot cases focus on a specific health condition, a particular population and setting, and a rehabilitation process that differs from the others.

The health conditions included, and the reasons for the rehabilitation approach, are:

1. People with hand and wrist disorders: this database contains a diverse set of common and rare hand and wrist disorders in adult patients collected in collaboration with Xpert Clinics in the Netherlands (10). Common pathologies include osteoarthritis in 1 or multiple joints, neuropathies such as carpal and cubital tunnel syndrome, Dupuytren’s disease, and wrist pathologies, including triangular fibrocartilage complex injuries. The database contains pre-treatment and post-treatment data of patients under either rehabilitation or surgical treatment. The most common treatment goals include pain reduction and (hand) function improvement during daily life (11).
2. Children with idiopathic scoliosis: idiopathic scoliosis is a deformity of the spine and trunk of unknown aetiology (12); during growth, rehabilitation aims to avoid progression that could cause health issues in adulthood, and specifically pain, plus mental health issues linked to the trunk deformity and progression, possibly leading to camptocormia with related problems in the elderly (13).
3. People with intermittent claudication: peripheral arterial disease (PAD) is a chronic disease mainly caused by atherosclerosis. Intermittent claudication (IC) is the most common symptom of PAD (14). IC is associated with limited walking distance and poor health-related quality of life. The primary objective of rehabilitation in patients with intermittent claudication entails addressing their specific needs, focusing on enhancing functional (walking) capacity, improving their ability to perform activities, and improving participation (15, 16). Concurrently, effective management also targets modifying risk factors associated with atherosclerosis.
4. People with lower limb loss: lower limb loss is a change in body structure comprising partial or complete amputation of 1 or both legs, below, through, or above the knee, caused primarily by vascular disease and diabetes, followed by trauma, cancer, and infection (17). It impacts all levels of people’s functioning (body functions, activities, and participation), in relation to personal factors (18). With a prosthesis, which is an environmental factor, many people can walk again and improve their overall health and functioning.
5. People with Parkinson’s disease or Parkinsonism: Parkinson’s disease and Parkinsonism constitute the second most prevalent neurodegenerative disorders worldwide. They impair independence in activities of daily living, restrict social participation, and diminish quality of life through a broad spectrum of motor symptoms (including bradykinesia, tremor, rigidity, dysarthria, dysphagia, gait disturbances, postural instability, and trunk abnormalities) (19), as well as non-motor symptoms (such as cognitive and mood disorders, psychosis, autonomic dysfunction including bladder and bowel disturbances, orthostatic hypotension, hyposmia, and pain) (20).
6. People who underwent total hip or knee replacement for primary or secondary osteoarthritis: primary osteoarthritis is a degenerative joint process caused by multiple biological and environmental factors (e.g., genetics, age, overweight, profession, natural wear and tear over time), leading to progressive levels of pain-related disability that cause, in turn, a downward spiral of muscle weakness, functional decline, and psychosocial health worsening; secondary osteoarthritis is still characterized by damage at the joint, but as a consequence of traumatic events and injuries (21); the latest stage of osteoarthritis is usually treated with joint replacement surgery, if the patient is eligible and willing to undergo major surgery; after surgery, global and segmental functional rehabilitation is needed (22, 23).
7. People with temporomandibular disorders: temporomandibular disorder (TMD) is a complex condition causing pain in the face, head, or neck, often involving the temporomandibular joint and masticatory muscles. Symptoms include joint pain, limited mouth opening, and clicking sounds, with the diagnosis sometimes remaining unclear despite clinical evaluation (24). Effective rehabilitation requires a comprehensive, individualized approach. Management typically involves multimodal therapy, including physical therapy, medication, occlusal splints, and behavioural strategies. Patient education and self-management, including stress reduction and jaw exercises, play a crucial role in promoting long-term recovery. Continuous monitoring and follow-up, aided by data collection tools, help track progress, adjust treatment, and prevent recurrence.

Table I lists the clinical partners of PREPARE and summarizes the main characteristics of their pilot cases.

**Table I:** List of clinical partners of PREPARE and the main characteristics of their pilot cases

*n*	Partner	Health condition	Population	Rehabilitation process
1	Erasmus MC, Universitair Medisch Centrum Rotterdam, the Netherlands	People with hand and wrist disorders	Adults	Outpatient splinting and physical, exercise, or occupational therapy The database also contains data from surgical interventions
2	ISICO (Istituto Scientifico Italiano Colonna Vertebrale), Milan, Italy	Children with idiopathic scoliosis	Children	Outpatient bracing and exercises
3	Radboud Universitair Medisch Centrum, Nijmegen, the Netherlands	People with intermittent claudication	Adults	Outpatient physical or exercise therapy
4	Univerzitetni Rehabilitacijski Institut Republike Slovenije-Soca, Liubljana, Slovenia	People with lower limb loss	Adults	Inpatient comprehensive rehabilitation and when appropriate prosthesis fitting
5	Università Politecnica delle Marche, Ancona, Italy	People with Parkinson’s disease or Parkinsonism	Adults (mostly elderly)	Outpatient neuromotor rehabilitation
6	IRCCS Ospedale Galeazzi Sant’Ambrogio, Milan, Italy	People who underwent hip and knee replacement for osteoarthritis	Adults (mostly elderly)	Inpatient gait, global and segmental function recovery
7	Università degli Studi di Milano, Milan, Italy	People with temporomandibular disorders	Adults	Outpatient exercise therapy

### Included databases

Distinct databases support each rehabilitation case. The main characteristics of each database are reported in Table II. Below, we describe each of them in more detail.

**Table II:** Main characteristics of the included databases

*n*	Database	Year of start of the database	Number of patients included
1	Erasmus MC, Universitair Medisch Centrum, Rotterdam, the Netherlands	2011	150,000
2	ISICO (Istituto Scientifico Italiano Colonna Vertebrale), Milan, Italy	2003	75,000
3	Radboud Universitair Medisch Centrum, Nijmegen, the Netherlands	2015	35,000
4	Univerzitetni Rehabilitacijski Institut Republike Slovenije-Soca, Liubljana, Slovenia	2021	400
5	Università Politecnica Delle Marche, Ancona, Italy	2007	1,132
6	IRCCS Ospedale Galeazzi Sant’Ambrogio, Milan, Italy	2023	2,110
7	Università degli Studi di Milano, Milan, Italy	2023	150

1. *Hand and wrist disorders:* The “Pulse” database has been operating in the Netherlands since 2011, aiming to enhance clinical care by providing feedback to clinicians on treatment outcomes and patient experiences and facilitating research. The content of the database has evolved. Initially, it included patient-reported outcomes and clinician-reported metrics such as grip strength and range of motion. Psychological questionnaires were integrated in 2017 to further personalize patient care. Subsequently, this database expanded to include preoperative screening tools and clinician dashboards.
2. *Idiopathic scoliosis in children:* The ScoliosisManager database, developed by ISICO in Italy in 2003, encapsulated all spinal deformity rehabilitation operations and research operations. The platform allows real-time data updates by healthcare providers and offers comprehensive modules for physicians (e.g., patient assessment, diagnosis, prescription management, X-ray data, storage of images and documents, brace sensor analysis, clinical questionnaires, and a clinical diary) and physical therapists (e.g., focuses on patient assessment, creating active self-correction movements, and formulating exercise plans). A mobile application introduced in 2016 has improved patient engagement by providing personalized feedback and educational content.
3. *Intermittent claudication:* The Chronic CareNet Quality system, established in 2015, collects data from primary care physiotherapy practices in the Netherlands. Variables included in the database were patient characteristics (e.g., age, sex, body mass index), treatment processes (e.g., treatment duration, number of treatment sessions, achievement of treatment goal), and patient-reported outcome measures (quality of life and walking distances). Predictive tools like KomPas facilitate shared decision-making by offering personalized outcome forecasts. The system’s integration with national guidelines ensures standardized treatment across healthcare providers.
4. *Lower limb loss:* A dedicated Excel database (Microsoft Corp, Armonk, NY, USA) initiated in 2021 in Slovenia tracks 417 variables across different professional groups, including physical and rehabilitation medicine, internal medicine, nursing, physiotherapy, occupational therapy, psychology, social work, and prosthetics. This database supports multidisciplinary collaboration and aims to identify predictors of rehabilitation outcomes, such as safe prosthetic fitting, and increased risk of falling and increased risk of wound development on the residual limb.
5. *Parkinson’s disease:* An electronic database developed since 2007 in Italy using Claris FileMaker® Pro records follow-up data for patients with Parkinson’s disease or Parkinsonism. During each visit, data were recorded on demographics, general health (including BMI and blood pressure values), disease and comorbidities history, medication schedule, functional surgery parameters, type and duration of rehabilitation treatment, complications, drug side effects, and standard assessments of disease severity (motor and cognitive). The database facilitates longitudinal tracking of disease progression and treatment efficacy, enabling the identification of key prognostic factors.
6. *Hip and knee replacement:* A patient-centred database for osteoarthritic patients undergoing inpatient rehabilitation after total hip or knee replacement was manually set for PREPARE. Preoperative data, surgical ward data, and rehabilitation ward data were linked by the principal investigator and researchers through the hospital patient identifier (MSP Trakcare). In this way, the entire inpatient pathway could be recomposed in search of potentially predictive factors, including pre-admission and post-discharge information already collected in the medical records (e.g., pre-existing comorbidities, pre-existing pharmacological treatment, demographic information, working status, living place, estimated hours of care needed after discharge, presence of a caregiver at home, potential frailty of the caregiver him/herself). Seventy-three variables were considered for each patient.
7. *Temporomandibular disorders:* A database developed in Italy in 2023–2024 includes diagnostic criteria, screening methods, and treatment protocols for TMD, with real-time data entry by clinicians. This database aims to improve the standardization of care and support research on treatment outcomes. It is mainly based on the diagnostic criteria for TMD, while adding information to address the recognised weaknesses of the Diagnostic Criteria–TMD, such as considering alternative structural or systemic causes and including a broader assessment of the muscles.

### Variables categorization

Variables were divided into 3 main categories:

1. *Outcomes:* Variables used to verify the results; more specifically, clinical outcomes that are measurable changes in health, symptoms, functioning, or quality of life resulting from the natural history or rehabilitation treatment.
2. *Modifiers:* Variables introduced due to treatment, i.e., therapeutic strategies that alter the progression or outcome of the health condition (e.g., exercises, drugs, surgery, or other strategies aimed at managing symptoms, improving functioning, preventing complications, or slowing the progression of the underlying condition). Also, other factors such as lifestyle changes (e.g., smoking cessation) or new comorbidities (e.g., heart disorders or diabetes mellitus) can be considered.
3. *Measurements:* Variables collected at baseline, before any treatment, such as demographic characteristics or quantified disease-related or functional data, serve as the basis for assessing potential improvements.

### Classifiers

We used the International Classification of Functioning, Disability and Health (ICF) (25) framework to classify the variables and enable comparison across all databases. According to the category of variables considered, it was necessary to either use or add further categorizers. We added patient reported outcome measures (PROMs) and discharge planning as outcome variables, as well as demographics, patient measures, laboratory data, risk factors, and costs for baseline measurements. Finally, for modifiers, we decided to use the following categories: rehabilitation, exercise therapy, orthoses, prostheses, physical modalities, other non-pharmacological and non-surgical interventions, drugs, surgery, prevention, medical history, and treatment timing.

### Data availability

All data supporting the findings of this study are included in the presentation. Additional details can be obtained from the corresponding author upon reasonable request.

## RESULTS

**Fig. 1:**
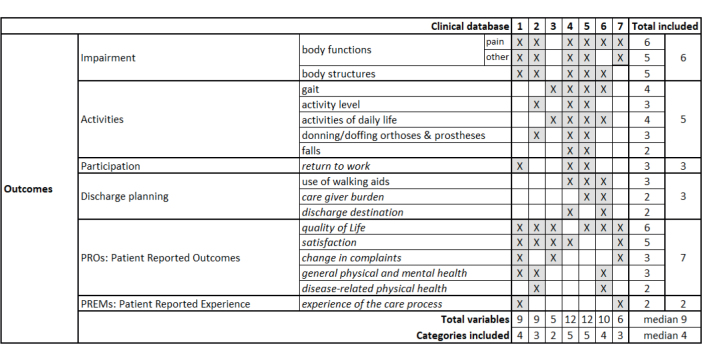
Outcome categories included in the considered databases. 1. Erasmus MC, Universitair Medisch Centrum, Rotterdam, the Netherlands; 2. ISICO (Istituto Scientifico Italiano Colonna Vertebrale), Milan, Italy; 3. Radboud Universitair Medisch Centrum, Nijmegen, the Netherlands; 4. Univerzitetni Rehabilitacijski Institut Republike Slovenije-Soca, Liubljana, Slovenia; 5. Università Politecnica delle Marche, Ancona, Italy; 6. IRCCS Ospedale Galeazzi Sant’Ambrogio, Milan, Italy; 7. Università degli Studi di Milano, Milan, Italy.

Fig. 1 reports the outcomes of the PREPARE databases. The social, behavioral and psychological indicators are reported in italics in this and the following figures. The databases included a median of 4 (range 2–5) of the 6 categories, with no database covering all categories. The most common categories were PROMs and impairments, included in 6 databases. Five databases contained data on some activities, but with differences in the types of activities considered. For participation, only the return-to-work component was included (3 databases). A median of 9 variables (range 6–12) was included. The most frequently measured variables were pain and quality of life (6 databases), followed by body structures and satisfaction (5 databases). We did not find any variables shared across all databases.

**Fig. 2:**
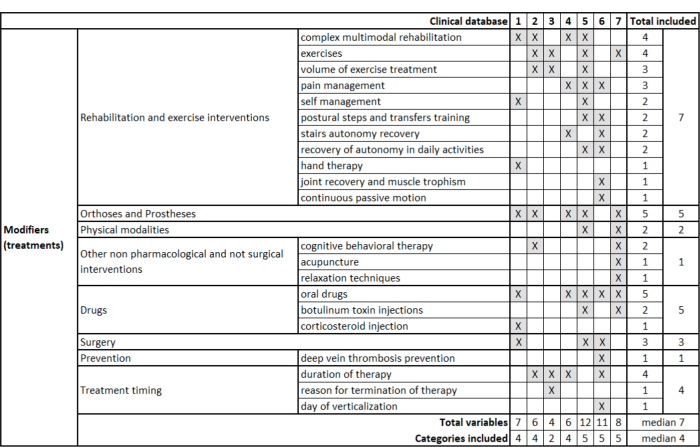
Modifiers (treatments) included in the considered databases. 1. Erasmus MC, Universitair Medisch Centrum, Rotterdam, the Netherlands; 2. ISICO (Istituto Scientifico Italiano Colonna Vertebrale), Milan, Italy; 3. Radboud Universitair Medisch Centrum, Nijmegen, the Netherlands; 4. Univerzitetni Rehabilitacijski Institut Republike Slovenije-Soca, Liubljana, Slovenia; 5. Università Politecnica delle Marche, Ancona, Italy; 6. IRCCS Ospedale Galeazzi Sant’Ambrogio, Milan, Italy; 7. Università degli Studi di Milano, Milan, Italy.

Fig. 2 shows the modifiers. The databases included a median of 7 modifiers (range 4–12) and considered 4 (2–5) of the 8 categories, with none considering all categories. The modifiers encompass various rehabilitation approaches, exhibiting notable variability. While all databases include rehabilitation or exercise approaches, the 2 most reported treatments are complex multimodal rehabilitation and exercises, each documented in 4 databases. The most frequently mentioned modifiers (present in 5 databases) are orthoses and prostheses and oral drugs, with variable therapy duration reported in 4 cases. All other variables appear in 3 or fewer databases.

**Fig. 3:**
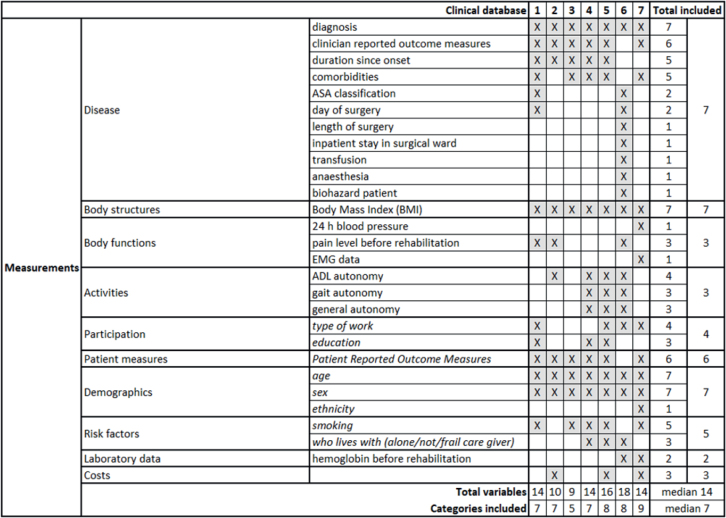
Baseline measurements included in the considered databases. 1. Erasmus MC, Universitair Medisch Centrum, Rotterdam, the Netherlands; 2. ISICO (Istituto Scientifico Italiano Colonna Vertebrale), Milan, Italy; 3. Radboud Universitair Medisch Centrum, Nijmegen, the Netherlands; 4. Univerzitetni Rehabilitacijski Institut Republike Slovenije-Soca, Liubljana, Slovenia; 5. Università Politecnica delle Marche, Ancona, Italy; 6. IRCCS Ospedale Galeazzi Sant’Ambrogio, Milan, Italy; 7. Università degli Studi di Milano, Milan, Italy.

Fig. 3 shows the measurements, mainly the variables at baseline assessment and all variables that are neither outcomes nor modifiers. The databases included a median of 14 measurements (range 9–18) and considered 7 (5–9) of the 10 categories, with no database including all of them. Among the categories, disease diagnosis, demographics (age and sex), and body structures (BMI) were included in all databases. PROMs were present at baseline in 6 of 7 databases; the duration of disease, comorbidities, and risk factors were included in 5 databases. Other measurements appeared in 4 or fewer cases.

Overall, we found 17 social, behavioral, and psychological indicators: quality of life, return to work, caregiver burden, discharge destination, satisfaction, change in complaints, general physical and mental health, disease-related physical health, experience of the care process, type of work, PROMs, education, age, sex, ethnicity, smoking. The least frequently reported information in the databases is caregiver burden and general physical and mental health (2 out of 7).

## DISCUSSION

The question we aimed to address in this paper was: Are there commonalities in the real-world collected data on rehabilitation for patients with various health conditions? Overall, we clearly see a trend of including similar categories across all databases. However, the most common categories remain those related to anthropometric, disease-specific, and impairment information. The “classical” biomedical approach appears to be more established (and includes more validated variables) than the biopsychosocial approach, at least in the databases considered. High importance is placed on patient-specific measures, both as outcomes and as baseline assessments. In contrast, core functioning data regarding activities and participation are less frequently considered. While similarities are evident, progress still needs to be made before achieving a truly standardized approach to data collection in rehabilitation across different health conditions.

Rehabilitation treatment is often complex and multimodal, involving multiple interventions simultaneously and an interdisciplinary multiprofessional approach (5). The 7 different databases analysed in this paper represent a significant portion of the rehabilitation field, even if musculoskeletal conditions are predominant. They differed significantly in the quantity and types of indicators collected, providing a broad view of the available outcomes, measurements, and therapy modifiers within rehabilitation. The findings highlight the variability in rehabilitation practices across various conditions, reflecting patients’ diverse needs and emphasizing the multimodal approach used. Our comparative analysis of 7 pilot cases representing the rehabilitation field revealed both common and unique features. Shared aspects include standardized data collection, validated outcome measures, and the inclusion of patient-reported outcomes. Variations were observed in the types of variables gathered, data entry methods, software programs used, and the extent to which databases are integrated into clinical practice. Additionally, the level of technological integration varied, with only some databases offering real-time dashboards and mobile applications that enhance clinician–patient interaction.

ICF is the common framework for rehabilitation (25). The use of the ICF as standardized common terminology has enabled meaningful comparisons between different cases. However, while the ICF served its purpose, it was unable to fully describe the included variables. In the era of big data, developing common vocabularies and data structures for clinical database development could be vital not only to better describe (and improve through research) clinical practices but also to allow for that database information to be combined for joint analysis. For example, while the ICF is a framework for common terminology, the same concepts defined by the same ICF terminology may still be coded differently in each database. Moreover, the ICF fails to provide terms for low-level concepts in rehabilitation at the level of the measurements or treatments (26), and does not define relationships between concepts. Initiatives like the multiprofessional Observational Health Data Sciences and Informatics (OHDSI) international community (27), promoted by PREPARE within the field of rehabilitation (8), could be instrumental in this regard. OHDSI adopted the Observational Medical Outcomes Partnership (OMOP) common data model (CDM) as a platform for standardizing the structure, content, and analytics of medical data. The OMOP-CDM is an open community data standard that enables efficient analyses to produce reliable evidence (28). A standardized framework to generate and evaluate patient-level prediction models using observational healthcare data has also been developed (29). It has been implemented as open-source software utilizing the OMOP-CDM to facilitate the sharing of models and the reproducibility of model evaluations across multiple observational datasets. A central component of the OMOP CDM is the OHDSI standardized vocabularies. The OMOP-CDM allows for standardized representation of the same data. Representing the same data across databases enables multicentre studies to be performed with standardized analytics that leverage the knowledge base within characterization, population-level effect estimation, and patient-level prediction studies.

While OMOP-CDM has been developed over many years and contains millions of medical concepts, we noticed that many typical rehabilitation concepts are still lacking in the current OHDSI vocabularies. Moreover, there is currently a limited possibility for structuring PROMs, PREMs, and complex longitudinal treatments in the existing OMOP-CDM domains. Therefore, some PREPARE partners joined forces to establish an OHDSI – Rehabilitation Workgroup (30). Such initiatives could encourage the inclusion of factors influencing the availability and utilization of effective care – such as patients’ income, insurance, culture, health literacy, as well as external factors related to healthcare providers (e.g., knowledge and beliefs), the healthcare system (e.g., staffing, wait times, incentives), and communities (e.g., rurality, transportation options, social supports). By promoting a standardized approach to data collection and analysis, the PREPARE project has the potential to transform rehabilitation practices across Europe and beyond.

The included databases reflect essential parts of the daily clinical practice of the partners involved. They were mainly developed to treat patients while collecting information for research purposes. To ensure real-world routine clinical data collection, decisions have been made to minimize the burden on physicians and gather useful information. As the primary focus is clinical, these databases were not designed to be “comprehensive” but to be “purpose-oriented”. Additionally, some of them were created many years ago and have gradually evolved into their current form, expanding data collection while keeping the workload manageable. This is a key characteristic of our study: not a depiction of what should be done, but of what is typically done.

Another important element to note is that the databases we explored are limited in number and do not allow for subgrouping. Nevertheless, they cover the entire field and represent a large number of patients across various areas of rehabilitation: inpatients, outpatients, rehabilitation and habilitation, acute, post-acute and chronic care, children, adults, and the elderly. It is worth noting that these databases are pioneers in rehabilitation, where the biopsychosocial approach makes data collection difficult. The limited number of databases could have contributed to the variability observed and, hypothetically, it is possible that within a specific area there could be more consistency in the rehabilitation of different health conditions. Nonetheless, this remains a hypothesis. In this regard, guiding health information systems to collect data for rehabilitation through routine health facility reporting built around a standard set of indicators (31) would provide a comprehensive view of the available outcomes, measurements, and therapy modifiers representative of the whole rehabilitation field and enhance the definition of rehabilitation sector targets, outcomes, and clinical decision-making across different health conditions.

### Strengths and limitations

This paper has several strengths and weaknesses. Strengths include the holistic approach to rehabilitation across various health conditions; international and multidisciplinary collaboration among 7 diverse groups; the use of the ICF framework for structured comparison; the inclusion of large, existing databases; the comprehensive qualitative process used to develop the report; and the representation of real-world clinical practice through variables applicable in everyday patient care. The weaknesses include the limited external validity of findings due to the small number of analysed databases and their heterogeneity, the underrepresentation of psychosocial and contextual factors, and a lack of advanced quantitative analyses. Additionally, some databases are recent or incomplete, which can affect the comprehensiveness of the data.

### Conclusion

This study demonstrates that rehabilitation databases across diverse health conditions share certain categories of information but remain heterogeneous and predominantly biomedical in focus. Psychosocial, contextual, and participation-related data are underrepresented, limiting a comprehensive understanding of the biopsychosocial nature of rehabilitation. Standardizing and expanding data collection will be essential to build robust, interoperable databases that can support personalized care and predictive modelling. Future efforts should prioritize increasing database inclusion, enhancing data standardization, and adopting rigorous statistical methods to refine personalized rehabilitation models and better capture the biopsychosocial complexity of patient care.
